# Robust estimation of the effect of an exposure on the change in a continuous outcome

**DOI:** 10.1186/s12874-020-01027-6

**Published:** 2020-06-06

**Authors:** Yilin Ning, Nathalie C. Støer, Peh Joo Ho, Shih Ling Kao, Kee Yuan Ngiam, Eric Yin Hao Khoo, Soo Chin Lee, E-Shyong Tai, Mikael Hartman, Marie Reilly, Chuen Seng Tan

**Affiliations:** 1 NUS Graduate School for Integrative Sciences and Engineering, National University of Singapore, 21 Lower Kent Ridge, Singapore, 119077 Singapore; 2 Yong Loo Lin School of Medicine, Department of Surgery, National University of Singapore and National University Health System, 10 Medical Dr, Singapore, 117597 Singapore; 3 Norwegian National Advisory Unit on Women’s Health, Oslo University Hospital, PO box 4950, Nydalen, 0424 Oslo, Norway; 4 Saw Swee Hock School of Public Health, National University of Singapore and National University Health System, 12 Science Drive 2, #10-01, Tahir Foundation Building, Singapore, 117549 Singapore; 5 Genome Institute of Singapore, 60 Biopolis St, Singapore, 138672 Singapore; 6 Yong Loo Lin School of Medicine, Department of Medicine, National University of Singapore and National University Health System, 10 Medical Dr, Singapore, 117597 Singapore; 7 University Medicine Cluster, Division of Endocrinology, National University Health System, 5 Lower Kent Ridge Rd, Singapore, 119074 Singapore; 8 University Surgical Cluster, Division of General Surgery (Thyroid and Endocrine Surgery), National University Health System, 5 Lower Kent Ridge Rd, Singapore, 119074 Singapore; 9 National University Health System Corporate Office, 5 Lower Kent Ridge Rd, Singapore, 119074 Singapore; 10 Cancer Science Institute of Singapore, National University of Singapore, 14 Medical Dr, Singapore, 117599 Singapore; 11 Department of Haematology-Oncology, National University Health System, 5 Lower Kent Ridge Rd, Singapore, 119074 Singapore; 12 Department of Medical Epidemiology and Biostatistics, Karolinska Institutet, PO Box 281, SE-171 77 Stockholm, Sweden

**Keywords:** Box-Cox transformation, Conditional probit model, Normal errors, Random effects model

## Abstract

**Background:**

The change in two measurements of a continuous outcome can be modelled directly with a linear regression model, or indirectly with a random effects model (REM) of the individual measurements. These methods are susceptible to model misspecifications, which are commonly addressed by applying monotonic transformations (e.g., Box-Cox transformation) to the outcomes. However, transforming the outcomes complicates the data analysis, especially when variable selection is involved. We propose a robust alternative through a novel application of the conditional probit (cprobit) model.

**Methods:**

The cprobit model analyzes the ordered outcomes within each subject, making the estimate invariant to monotonic transformation on the outcome. By scaling the estimate from the cprobit model, we obtain the exposure effect on the change in the observed or Box-Cox transformed outcome, pending the adequacy of the normality assumption on the raw or transformed scale.

**Results:**

Using simulated data, we demonstrated a similar good performance of the cprobit model and REM with and without transformation, except for some bias from both methods when the Box-Cox transformation was applied to scenarios with small sample size and strong effects. Only the cprobit model was robust to skewed subject-specific intercept terms when a Box-Cox transformation was used. Using two real datasets from the breast cancer and inpatient glycemic variability studies which utilize electronic medical records, we illustrated the application of our proposed robust approach as a seamless three-step workflow that facilitates the use of Box-Cox transformation to address non-normality with a common underlying model.

**Conclusions:**

The cprobit model provides a seamless and robust inference on the change in continuous outcomes, and its three-step workflow is implemented in an R package for easy accessibility.

## Background

In studies with repeated measurements of a continuous outcome, it may be of interest to quantify the change in the outcome over time. For example, the effect of a treatment can be assessed by comparing the outcome measured for each subject before and after receiving the treatment in a pre-post study [1, 2]. In experimental studies under well-controlled settings, the simple paired t-test may be sufficient for assessing the change in a continuous outcome at two time points in each subject. To control for confounding when assessing these changes in observational studies, we could generalize the paired t-test to a linear regression model. However, the validity of the inference from the regression model relies on the adequacy of model assumptions, i.e., normality or linearity.

Transformation is commonly used when residual diagnostics reveal that the model assumptions are inadequate. The commonly considered Box-Cox power family [3] is defined for positive values, and so cannot be applied to the change in continuous measurements with negative differences. In these situations, the shifted power family [3] can be considered, but the likelihood function of the transformation parameter may behave poorly, resulting in the standard asymptotic properties of the maximum likelihood estimator being invalid [4, 5]. Although the Yeo-Johnson method of transformation [6] is well-defined for both positive and negative values, it produces estimates that are difficult to interpret, due to the different powers for the transformation of positive and negative values [7]. Given these difficulties, it may be preferable to apply the Box-Cox transformation on the individual measurements.

An alternative approach is to analyze the repeated measurements by using the random effects model (REM) with a random intercept for each subject [8], and address the inadequacy of the normality assumption using the Box-Cox transformation [9]. However, different transformations selected for the same outcome variable in different studies may result in conflicting findings [10] and could complicate the selection of variables in the final model: the independent variable(s) selected at each step of the model-building procedure may differ from those that would have been selected from modelling the untransformed data, due to the additional (re)estimation of the transformation parameter at each step [11]. Hence, it is appealing to devise a robust analytical approach that allows inference on the effect of the predictor that is indifferent to any monotonic transformation on the outcome.

To address the issues faced with transformation in studies of independent samples, Liu and team [10] and Tan and team [12] proposed to analyze the ordering of outcomes where the resulting exposure effect is invariant to monotonic transformations. In particular, Tan and team introduced a stratified analysis of continuous outcomes for confounder adjustment, where the strata consisted of subjects with the same or similar confounding profile. This approach can be adapted in the context of two repeated outcome measurements by considering each subject as a stratum: assuming normally distributed error terms, the two ordered outcome measurements can be analyzed using the conditional probit (cprobit) model, allowing robust inference on the presence and direction of an exposure effect.

In this paper, we propose a robust approach to analyze two repeated measurements of a continuous outcome, by first applying the cprobit model to detect an exposure effect and subsequently quantifying the exposure effect on the observed outcome. We compare the performance of our proposed approach to the REM using simulated data, and provide a seamless three-step workflow for assessing exposure effect that facilitates the use of Box-Cox transformation to address non-normality with a common underlying model. We illustrate the application of our proposed approach with two real datasets which utilize electronic medical records (EMRs): a study of the association between the changes in white blood cell count and neutrophil percentage in female breast cancer patients after initiating chemotherapy; a study of glycemic variability over a three-day period among hospitalized patients, to determine the association between the baseline values and the change in the subsequent two days.

## Methods

### Underlying random effects model

We consider two repeated measurements of a continuous exposure (*x*_*ij*_) and a continuous outcome (*y*_*ij*_) from the *i*-th subject, *i* = 1, …, *n* and *j* = 1, 2, and assume the observed outcome is generated from the following REM:

1 $$ {y}_{ij}={\alpha}_i+\beta {x}_{ij}+{\varepsilon}_{ij}, $$

where *ε*_*ij*_~*N*(0, *σ*^2^) denotes the independent measurement error within each subject, and the random intercept ${\alpha}_i\sim N\left({\mu}_{\alpha },{\sigma}_{\alpha}^2\right)$ represents the subject-specific time-invariant effects that is assumed to be independent from *ε*_*ij*_ and *x*_*ij*_ [8]. Departure from the normality assumptions could be assessed using the simple residuals [8], and if the normality assumption is inadequate, the Box-Cox transformation can be applied to the outcome to achieve a normal distribution for the ‘total’ error term [9].

### Difference model

The parametric assumption on the intercept terms makes the REM susceptible to model misspecification. An alternative approach to make inference on *β* is to apply the linear regression model to the change in the outcome within each subject from equation (1):

2 $$ \Delta  {y}_{i.}=\beta \Delta  {x}_{i.}+\Delta  {\varepsilon}_{i.}, $$

where *∆y*_*i*._ = *y*_*i*2_ − *y*_*i*1_, *∆x*_*i*._ = *x*_*i*2_ − *x*_*i*1_, and $\Delta  {\varepsilon}_{i.}={\varepsilon}_{i2}-{\varepsilon}_{i1}\sim N\left(0,{\sigma}_{\Delta }^2=2{\sigma}^2\right)$ are scalar quantities. This differencing approach eliminates the subject-specific intercept and thereby avoids the assumptions of normality on it [8].

### Conditional probit model for continuous outcomes

Similar to non-parametric statistical tests, the cprobit model uses the ordering of the outcomes within each subject to perform hypothesis testing on *β*. It is derived from the scaled difference from equation (2) where the new error term has unit variance [13]:

3 $$ \Delta  {y}_{i.}/{\sigma}_{\Delta  }={\beta}_c\Delta  {x}_{i.}+\Delta  {\varepsilon}_{i.}/{\sigma}_{\Delta  }, $$

where *β*_*c*_ = *β*/*σ*_*∆*_. Hence the probability of observing *y*_*i*2_ > *y*_*i*1_ (or equivalently *∆y*_*i*._/*σ*_*∆*_ > 0) for the *i*-th subject is:

4 $$ \mathit{\Pr}\left({I}_i=1\right)=\mathit{\Pr}\left(\Delta  {y}_{i.}/{\sigma}_{\Delta  }>0\right)=\varPhi \left({\beta}_c\Delta  {x}_{i.}\right), $$

where *I*_*i*_ = *I*{*y*_*i*2_ > *y*_*i*1_} and Φ(·) is the cumulative density function of a standard normal distribution. The estimate for *β*_*c*_ (${\hat{\beta}}_c$) can be used to assess the presence and direction of an association between the exposure and the outcome.

### Estimation of linear effect and residuals for conditional probit model

As the parameter of interest is the linear exposure effect on the continuous outcome (*β*) and *β*_*c*_ = *β*/*σ*_*∆*_ from equation (3), we adapt the approach proposed by Tan and team [12] to estimate *β* given ${\hat{\beta}}_c$ by rewriting equation (2) as:

5 $$ \Delta  {y}_{i.}={\sigma}_{\Delta  }{\beta}_c\Delta  {x}_{i.}+\Delta  {\varepsilon}_{i.}, $$

and we estimate *σ*_*∆*_ by maximizing the estimated likelihood [14] based on equation (5), where *β*_*c*_ is replaced by ${\hat{\beta}}_c$. With estimates available for both ${\hat{\beta}}_c$ and ${\hat{\sigma}}_{\Delta  }$, the residuals in equation (5) correspond to ${\hat{\Delta  \varepsilon}}_{i.}=\Delta  {y}_{i.}-{\hat{\sigma}}_{\Delta  }{\hat{\beta}}_c\Delta  {x}_{i.}$ and are subsequently used to assess the adequacy of the model assumptions. If the normality assumption is adequate, *β* can be estimated using the plug-in estimator: $\hat{\beta}={\hat{\sigma}}_{\Delta  }{\hat{\beta}}_c$, with standard error: $SE\left(\hat{\beta}\right)={\hat{\sigma}}_{\Delta  } SE\left({\hat{\beta}}_c\right)$.

### Addressing model inadequacy with Box-Cox transformation

#### Modelling the transformed outcome

When the normality assumption on *∆ε*_*i*._ is inadequate, we consider a Box-Cox transformation (indexed with a parameter *λ*) of the observed outcome *y*_*ij*_:

$$ {y}_{ij}^{\left(\lambda \right)}=\left\{\begin{array}{ll}\left({y}_{ij}^{\lambda }-1\right)/\lambda & \mathrm{if}\ \lambda \ne 0\\ {}\log \left({y}_{ij}\right)& \mathrm{if}\ \lambda =0.\end{array}\right. $$

We assume ${y}_{ij}^{\left(\lambda \right)}$ can be described by the following REM:

6 $$ {y}_{ij}^{\left(\lambda \right)}={\alpha}_{\lambda i}+{\beta}_{\lambda }{x}_{ij}+{\varepsilon}_{\lambda i j}, $$

where ${\varepsilon}_{\lambda ij}\sim N\left(0,{\sigma}_{\lambda}^2\right)$ and ${\alpha}_{\lambda i}\sim N\left({\mu}_{\lambda \alpha},{\sigma}_{\lambda \alpha}^2\right)$, and the subscript *λ* indicates the dependency of the parameters on *λ*. When *λ* = 1, equation (6) is simply the linear model on the untransformed outcome *y*_*ij*_ (i.e., equation (1) where *α*_*λi*_ = *α*_*i*_ − 1, *β*_*λ*_ = *β* and *ε*_*λij*_ = *ε*_*ij*_). Similar to the analysis on the untransformed outcome, we eliminate the subject-specific intercept by working on the difference in the transformed outcomes within each subject:

7 $$ \Delta  {y}_{i.}^{\left(\lambda \right)}={\beta}_{\lambda}\Delta  {x}_{i.}+\Delta  {\varepsilon}_{\lambda i.}, $$

where $\Delta  {y}_{i.}^{\left(\lambda \right)}={y}_{i2}^{\left(\lambda \right)}-{y}_{i1}^{\left(\lambda \right)}$, *∆x*_*i*._ = *x*_*i*2_ − *x*_*i*1_, and $\Delta  {\varepsilon}_{\lambda i.}={\varepsilon}_{\lambda i2}-{\varepsilon}_{\lambda i1}\sim N\left(0,{\sigma}_{\lambda \Delta }^2=2{\sigma}_{\lambda}^2\right)$ are scalar quantities.

#### Conditional probit model for transformed outcome

The cprobit model for the transformed outcome can be derived from the scaled difference of the transformed outcome in equation (7):

8 $$ \Delta  {y}_{i.}^{\left(\lambda \right)}/{\sigma}_{\lambda \Delta  }={\beta}_{c\lambda}\Delta  {x}_{i.}+\Delta  {\varepsilon}_{\lambda i.}/{\sigma}_{\lambda \Delta  }, $$

where *β*_*cλ*_ = *β*_*λ*_/*σ*_*λ∆*_ and *∆ε*_*λi*._/*σ*_*λ∆*_~*N*(0, 1). Since the Box-Cox transformation does not change the ordering of the outcome, equation (4) can be rewritten as:

9 $$ \mathit{\Pr}\left({I}_i=1\right)=\mathit{\Pr}\left(\Delta  {y}_{i.}/{\sigma}_{\Delta  }>0\right)=\mathit{\Pr}\left(\Delta  {y}_{i.}^{\left(\lambda \right)}/{\sigma}_{\lambda \Delta  }>0\right)=\varPhi \left({\beta}_{c\lambda}\Delta  {x}_{i.}\right), $$

where the estimate of *β*_*cλ*_ is again ${\hat{\beta}}_c$ and Φ(·) is the cumulative density function of a standard normal distribution. To estimate *β*_*λ*_ given ${\hat{\beta}}_c$ requires the estimation of *λ* and *σ*_*λ∆*_. Following from equation (5), we use the relationship *β*_*λ*_ = *σ*_*λ∆*_*β*_*cλ*_ to rewrite equation (7) as:

10 $$ \Delta  {y}_{i.}^{\left(\lambda \right)}={\sigma}_{\lambda \Delta  }{\beta}_{c\lambda}\Delta  {x}_{i.}+\Delta  {\varepsilon}_{\lambda i.}. $$

We follow the common practice [3, 5] to estimate *λ* and *σ*_*λ∆*_ by maximizing the profile likelihood. If residual diagnostics using ${\hat{\Delta  \varepsilon}}_{\lambda i.}=\Delta  {y}_{i.}^{\left(\hat{\lambda}\right)}-{\hat{\sigma}}_{\lambda \Delta  }{\hat{\beta}}_c\Delta  {x}_{i.}$ support the normality assumption, we estimate *β*_*λ*_ using the plug-in estimator: ${\hat{\beta}}_{\lambda }={\hat{\sigma}}_{\lambda \Delta  }{\hat{\beta}}_c$, with standard error: $SE\left({\hat{\beta}}_{\lambda}\right)={\hat{\sigma}}_{\lambda \Delta  } SE\left({\hat{\beta}}_c\right)$.

### Three-step workflow

**Fig. 1:**
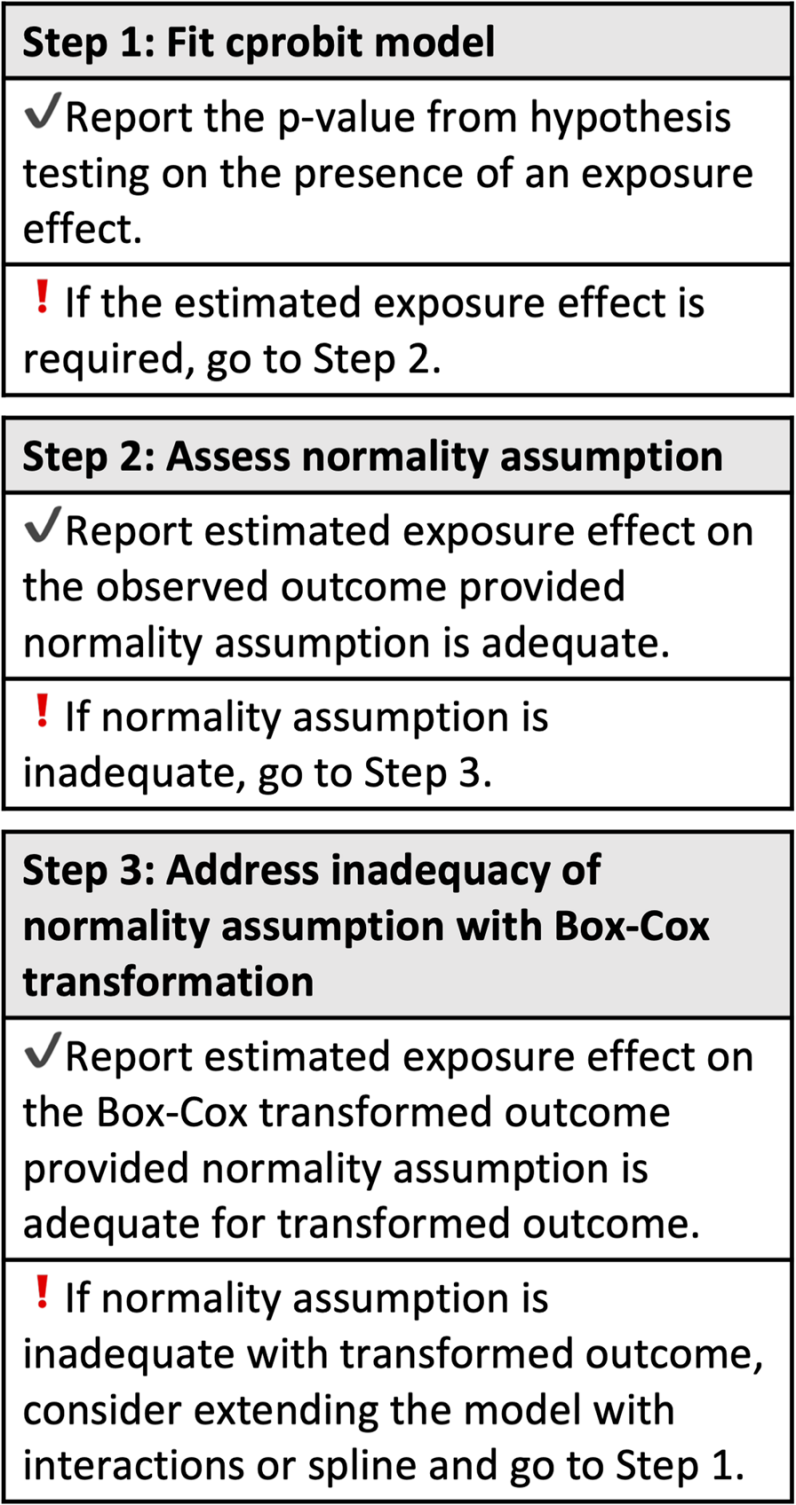
Illustration of the three-step workflow of the cprobit model for the analysis of continuous outcomes

Our proposed approach seamlessly integrates the use of Box-Cox transformation to alleviate non-normality, which we summarize as a three-step workflow (see Fig. 1). Assuming the (transformed) outcome is generated from a REM, our approach estimates the exposure effect by working on the differences in the (transformed) outcomes from each subject. In Step 1 of the workflow, the presence and direction of this effect is assessed by applying the cprobit model to model the indicator *I*_*i*_ in equation (4) of the positive difference in the observed outcomes within each subject (see Supplementary Figure S1 in Additional file 1 for a detailed summary of the three-step workflow). The estimated effect from the cprobit model, ${\hat{\beta}}_c$, is unchanged when a Box-Cox transformation is applied to the outcome (see equation (9)), and it is scaled in the subsequent steps to quantify the linear exposure effect on the (transformed) outcome.

In Step 2, we first consider a simple scenario where the observed outcome is generated from a REM (see equation (1)). Although our approach works on the differences in the observed outcomes within each subject (see equation (2)), ${\hat{\beta}}_c$ provides an estimate for the scaled exposure effect on the observed outcome, where the scaling factor is the reciprocal of the standard deviation of the error distribution of the differences (see equation (3)). Thus, if the residual diagnostics suggest the error terms *∆ε*_*i*._ in equation (2) are normal, the estimated exposure effect on the observed outcome is: $\hat{\beta}={\hat{\sigma}}_{\Delta  }{\hat{\beta}}_c$.

When the normality assumption on the error term is inadequate for the observed outcome in Scenario 1, scaling ${\hat{\beta}}_c$ from Step 1 may not be appropriate. We address this issue with the Box-Cox transformed outcome where a REM is assumed for the transformed outcome (see equation (6)), and our approach estimates the exposure effect using the difference in the transformed outcomes (see equation (7)). The transformation parameter (*λ*) is estimated and the ${\hat{\beta}}_c$ from Step 1 is now the estimate of the scaled effect on the transformed outcome (see equation (8)). The estimated effect on the transformed outcome is: ${\hat{\beta}}_{\lambda }={\hat{\sigma}}_{\lambda \Delta  }{\hat{\beta}}_c$. When the Box-Cox transformation in Step 3 is insufficient to alleviate non-normality, it may be useful to consider extensions of the functional relationship between the exposure and the outcome, e.g., by including interaction terms or splines.

### Simulation study

#### Simulation study 1

This study assessed and compared the performance of the REM and the cprobit model when assessing the association between a predictor at baseline and the change in the outcome without transformation (see Additional file 2 for detailed simulation setup). To investigate the performance of the two methods under different conditions, we simulated data with different effect sizes of the predictor (*β* = 0 and −0.06), sample sizes (*n* = 300 and 1200) and random intercept distributions (normal and skewed). We assessed the performance of the two methods from 2000 simulation cycles using the type I error, power, bias, empirical standard error (empirical SE), average of the model-based standard error (mean SE) and the coverage of the 95% confidence intervals (CIs) extracted for the estimated linear effect ($\hat{\beta}$).

#### Simulation study 2

This study assessed and compared the performance of the two methods when the Box-Cox transformation on the outcome provides a linear model with normal errors. To generate such an observed continuous outcome, we used the outcome generated in Simulation study 1, and applied the inverse Box-Cox transformation with *λ* = 1, 1/3, 0 to obtain the observed continuous outcome prior to the (supposedly unknown) transformation. Thus the created data required no transformation, a cubic root transformation and a log transformation respectively to satisfy the normality assumption. We assessed the performance of the estimated effect on the transformed outcome (${\hat{\beta}}_{\lambda }$) and the estimated transformation parameter ($\hat{\lambda}$) from the two methods, and assessed the adequacy of the normality assumption after the Box-Cox transformation with the Lilliefors test [15] on the residuals.

### Data illustrations

We used two real datasets which utilize EMRs to illustrate our proposed workflow and compared it with: (i) the conventional linear regression model when the normality assumption is adequate without Box-Cox transformation, and (ii) the REM with or without transformation as appropriate.

#### Neutrophil study

We investigated the association between the change in white blood cell (WBC) counts and the change in the neutrophil percentage before and during chemotherapy among breast cancer patients, where a low neutrophil level is a risk indicator for developing severe side effects of chemotherapy [16]. This study used retrospective data from breast cancer patients diagnosed between 2005 and 2014, who underwent chemotherapy at the National University of Singapore (NUH). The two periods considered in this study were the 60 days before (period 1) and after (period 2) the start date of chemotherapy. We focused our investigation on 384 patients who did not require medical intervention to increase neutrophil counts during these periods [17], with diagnostic information retrieved from the NUH breast cancer registry and laboratory test results from the NUH EMRs. For each patient, we extracted the minimum WBC count and minimum neutrophil percentage for each period, and subsequently dichotomized the minimum WBC count in each period using the sample median in period 1 (6.94 × 10^9^/*L*).

We assumed the following REM:

11 $$ {y}_{ij}={\alpha}_i+{\beta}_1{x}_{ij}+{\beta}_2{t}_{ij}+{\beta}_3{Age}_i+{\beta}_4{Stage}_i+{\beta}_5{Ethnicity}_i+{\varepsilon}_{ij}, $$

where *y*_*ij*_ represents the minimum neutrophil percentage for the *i*-th subject in the *j*-th period, *x*_*ij*_ indicates whether the minimum WBC count for the *i*-th subject in the *j*-th period is higher than the cut-off defined earlier, *t*_*ij*_ is the indicator for period 2, Age_*i*_ represents age at diagnosis, Stage_*i*_ indicates stage 3 or above and Ethnicity_*i*_ indicates Chinese, for *i* = 1, …, 384, and *j* = 1, 2. As the linear regression and the cprobit models are applied to the difference in outcomes, these time-invariant covariates are implicitly controlled for.

#### Blood glucose study

High glycemic variability is a known risk factor for diabetes complications [18, 19]. We examined whether the baseline glycemic variability was associated with the change in subsequent daily glycemic variability by using the point-of-care capillary blood glucose (BG) readings collected from the first BG monitoring episode among hospitalized non-critical care adult patients in NUH, using data retrieved from the NUH EMRs. We defined a BG monitoring episode as a contiguous sequence of BG readings where consecutive readings were no more than two days apart. For each patient, the daily glycemic variability was quantified by the standard deviation (SD) of the BG readings. The baseline variability is represented by the SD on the first day of the monitoring episode, and the change in the SD between the second and third day of the same episode (referred to as the first and second follow-up) is the outcome of primary interest. We included patients warded in the medical wards between September and December in 2012 if their first BG monitoring episode was at least three days in duration and at least three BG readings were collected per day during the first three days of the episode. The final dataset included 1200 patients.

We assumed the following REM:

12 $$ {y}_{ij}={\alpha}_i+{\beta}_1{t}_{ij}+{\beta}_2{SD}_{0i}+{\beta}_3{t}_{ij}{SD}_{0i}+{\beta}_4{Age}_i+{\beta}_5{Female}_i+{\varepsilon}_{ij}, $$

where *y*_*ij*_ represents the SD of BG for the *i*-th subject in the *j*-th follow-up, *t*_*ij*_ is an indicator for the second follow-up, *SD*_0*i*_ represents the baseline SD of BG, Age_*i*_ represents age at baseline and Female_*i*_ indicates female for *i* = 1, …, 1200, and *j* = 1, 2. By taking the difference between *y*_*i*1_ and *y*_*i*2_ in equation (12), we obtain the following linear model:

13 $$ \Delta  {y}_{i.}={\beta}_1+{\beta}_3{SD}_{0i}+\Delta  {\varepsilon}_{i.}. $$

Equation (13) suggests that *β*_3_ can be interpreted as the linear effect of the baseline variability of BG on the change in variability of BG between the first and second follow-up of the monitoring episode, and the time-invariant covariates are implicitly controlled for when the linear regression and the cprobit models are applied to the difference in outcomes.

### Implementation

The analyses were performed using R version 3.2.3 [20]. The *lmer* function in the *lme4* package [21] was used to implement the REM, and the *powerTransform* function in the *car* package [22] was used to estimate *λ* when the Box-Cox transformation was used. The cprobit model in Step 1 of our workflow was implemented using the *glm* function by specifying the binomial family for *I*_*i*_ with probit link. To obtain $\hat{\lambda}$ in Step 3, the profile likelihood was maximized using the *optimize* function. Since it is common practice to restrict *λ* to take values from a closed interval [7, 23], we follow Hawkins and Weisberg [23] by considering the interval [−3, 3]. We implemented the proposed three-step workflow as an R package named *cprobit* [24] (see Additional file 3 for a reproducible example). Residual diagnostics was performed in Step 2 and 3 of our workflow using the *lillie.test* function in the *nortest* package [25].

## Results

### Simulation study

#### Simulation study 1: without Box-Cox transformation

**Fig. 2:**
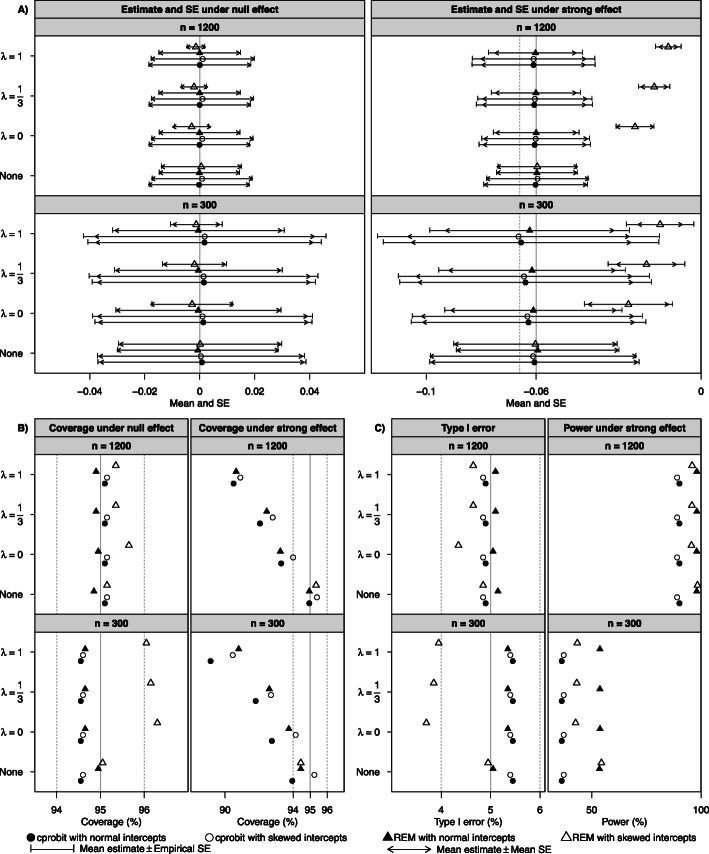
Performance of the REM and cprobit model in estimating the linear effect in simulation studies. Mean and standard error (panel **a**), coverage (panel **b**) and type I error and power (panel **c**) of the estimated linear effect under null and strong effects from the random effects model (REM) and the conditional probit (cprobit) model when applied to the scenarios where no transformation was required (“None”) and Box-Cox transformation was considered ($\lambda =0,\frac{1}{3},1$), with normal and skewed intercept terms, small and large sample sizes (*n* = 300, 1200). Solid vertical grey lines indicate the true effect sizes in panel **a**, and the nominal value of the coverage and type I error in panel **b** and **c**. Dashed vertical grey lines indicate a 10% bias in the estimate under the strong effect in panel A, and ±1% deviation from the nominal values in panel B and C. (Note: Under strong effect, the coverage of the REM with skewed intercepts was 34.2% or lower for $\lambda =0,\frac{1}{3},1$ and beyond the plot range for panel **b**)

Both models provided unbiased estimates ($\hat{\beta}$) with comparable mean SE and empirical SE, type I error close to 5% and coverage close to 95% for the various settings considered (see the panels labelled “None” in Fig. 2 and Supplementary Table S1 in Additional file 1). The $\hat{\beta}$ from the cprobit model had larger SE and lower power than that from the REM.

#### Simulation study 2: with Box-Cox transformation

**Fig. 3:**
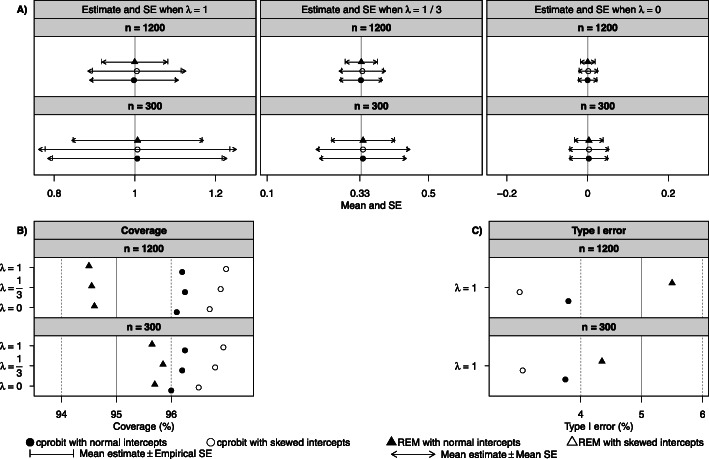
Performance of the REM and cprobit model in estimating the transformation parameter in simulation studies. Mean and standard error (panel **a**), coverage (panel **b**) and type I error (panel **c**) of the estimated transformation parameter ($\hat{\lambda}$) from the random effects model (REM) and the conditional probit (cprobit) model with the Box-Cox transformation for strong effect (*β*_*λ*_ =  − 0.06), with $\lambda =0,\frac{1}{3},1$, normal and skewed intercept terms, *n* = 300, 1200. Solid vertical grey lines indicate the true *λ* values in panel **a**, and the nominal value of the coverage and type I error in panel **b** and **c**. Dashed vertical grey lines indicate ±1% deviation from the nominal values in panel **b** and **c**. (Note: Results for the REM with skewed intercepts are beyond the plot range: (**a**) $\hat{\lambda}<0.1$ when *λ* = 1, $\hat{\lambda}<-0.1$ when *λ* = 1/3, and $\hat{\lambda}<-0.19$ when *λ* = 0 for panel A; (**b**) coverage at 5.5% or below for panel **b**; and (**c**) type I error at 94.5% or above for panel **c**)

We first summarize the performance of both methods when the intercepts were normally distributed. Simulation results of the estimate (${\hat{\beta}}_{\lambda }$) are summarized in Supplementary Table S2 in Additional file 1 and illustrated in the panels labelled *λ* = 0, 1/3 and 1 in Fig. 2. For zero effect with both sample sizes, both the cprobit model and the REM with Box-Cox transformation produced unbiased ${\hat{\beta}}_{\lambda }$ with comparable mean SE and empirical SE, coverage close to 95% and therefore type I error close to 5%. For non-zero effect (*β*_*λ*_ =  − 0.06), the estimates from both methods were unbiased when sample size was large (*n* = 1200) but became somewhat biased with small sample size (*n* = 300), especially for larger *λ* values. Although the bias was slightly larger from the cprobit model than the REM, it was generally within 10% of the true value of *β*_*λ*_. The coverage was slightly lower than 95% for non-zero effect when *λ* = 0 in both sample sizes and methods due to some underestimation of the standard error, which was more pronounced for larger *λ*. As observed in Simulation study 1, the REM had higher power than the cprobit model. The estimated transformation parameter ($\hat{\lambda}$) from both methods had good inferential properties except for the slightly conservative type I error and coverage from the cprobit model (see Fig. 3 and Supplementary Table S3 in Additional file 1). The rate of rejecting the normality assumption after the Box-Cox transformation was close to the expected level of 5% for both methods (see Supplementary Table S4 in Additional file 1).

The performance of the cprobit model with Box-Cox transformation was not affected by the skewed distribution of the intercept terms, but the REM with Box-Cox transformation was adversely affected: it failed to address non-normality in many simulation cycles especially when *n* = 1200 (see the high rejection rate in Supplementary Table S4 in Additional file 1), resulting in biased estimate and low coverage when *β*_*λ*_ =  − 0.06, and conservative type I error when *n* = 300 and lower power than for normal intercepts.

### Real data analyses

#### Neutrophil study

The residual qq-plot and the Lilliefors test suggested the adequacy of the normality assumption for the linear regression model applied to the changes in the outcome (see Supplementary Figure S2(A) in Additional file 1), and hence we analyzed the observed outcome without transformation. When the WBC status changed from low in period 1 to high in period 2, the linear regression model estimated a significant increase in the minimum neutrophil percentage (i.e., expected increase is 10.20, 95% CI: 8.44, 11.95; see Table 1). Residual diagnostics for the REM and the cprobit model also supported the reporting of estimated effect for the untransformed data (see Supplementary Figure S2(B) and S2(C) in Additional file 1). Estimates of linear effect from both methods were consistent with the linear regression model: 9.51 (95% CI: 8.15, 10.88) from the REM, and 11.29 (95% CI: 8.66, 13.92) from the cprobit model.

**Table 1:** Results from the neutrophil study

**Method**	**Linear effect on untransformed outcome**	
	**Estimate**	**95% Confidence interval**
Linear regression^a,b^	10.20	8.44, 11.95
REM^c^	9.51	8.15, 10.88
cprobit^b^	11.29	8.66, 13.92

^a^Linear regression model on the change in outcomes

^b^Implicitly adjusted for all time-invariant confounders

^c^Adjusted for age, stage and ethnicity by including these variables into the linear predictor

#### Blood glucose study

Both the residual qq-plot and the Lilliefors test suggested the inadequacy of the normality assumption when applying the linear regression model to the changes in the untransformed outcome (see Supplementary Figure S3 in Additional file 1), and a similar conclusion was drawn from residual diagnostics of the REM and the cprobit model (see Supplementary Figure S4(A) and S4(B) in Additional file 1). Therefore, the Box-Cox transformation was used in the REM and the cprobit model in the subsequent analysis to address non-normality. Both models identified a need to transform ($\hat{\lambda}=0.33$ for the REM and $\hat{\lambda}=0.34$ for the cprobit, see Table 2), and the residual qq-plots and the Lilliefors test suggested the adequacy of the normality assumption after transformation (see Supplementary Figure S4(C) and S4(D) in Additional file 1). Residual diagnostics suggested that the REM had a better fit than the cprobit model for analyzing the transformed data, although both models generated similar estimates for the linear effect of the baseline measurement on the subsequent change in the transformed outcome: − 0.054 (95% CI: − 0.083, − 0.025) from the REM and − 0.042 (95% CI: − 0.079, − 0.005) from the cprobit model. A simulated dataset based on this study is provided in the *cprobit* package. The R commands to analyze this simulated dataset using the three-step workflow and their output are documented in Additional file 3.

**Table 2:** Results from the blood glucose study

**Method**	**Linear effect on transformed outcome**		**Transformation parameter (*λ*)**	
	**Estimate**	**95% Confidence interval**	**Estimate**	**95% Confidence interval**
REM^a^	–0.054	−0.083, −0.025	0.33	0.28, 0.37
cprobit^b^	−0.042	−0.079, −0.005	0.34	0.28, 0.40

^a^Adjusted for age and gender by including these variables into the linear predictor

^b^Implicitly adjusted for all time-invariant confounders

## Discussion

In this paper, we have proposed a robust alternative to simple linear regression or REM for the analysis of change in two repeated measurements of a continuous outcome. Our method involves a novel application of the cprobit model that incorporates the Box-Cox transformation. By modeling the change, the cprobit model eliminates the subject-specific intercept after taking the difference, and is therefore less susceptible to model misspecification than the REM. Simulation studies highlighted the advantage of the cprobit over the REM when the Box-Cox transformation was required. Based on the framework of our proposed approach, we described a three-step workflow, and applied it to two real datasets that utilize EMRs to illustrate the different situations that can arise in practical data analysis.

Findings from our simulation study demonstrated a good performance for both the REM and the cprobit model when no transformation was required, although the estimates from the cprobit model had higher variability (and hence lower power) than REM. With the Box-Cox transformation and normally distributed intercept terms, both methods provided good estimates of the linear exposure effect on the transformed outcome (${\hat{\beta}}_{\lambda }$) for zero effect, and the power of both methods was not affected by the need to estimate the transformation parameter (*λ*) for both effect sizes. However, for non-zero effects, an underestimation of the SE of ${\hat{\beta}}_{\lambda }$ from both methods resulted in reduced coverage, and for small sample size (*n* = 300) a bias, that was generally smaller for REM, despite the estimated transformation parameter ($\hat{\lambda}$) being unbiased. The bias and underestimated variability of ${\hat{\beta}}_{\lambda }$ are attributable to the uncertainty in $\hat{\lambda}$ that is not accounted for in estimating *β*_*λ*_ [9, 26]. Consistent with the literature [27] the REM without transformation was robust against a skewed distribution of the intercept terms. However, the REM with Box-Cox transformation may fail to overcome the non-normality in the ‘total’ error term [9] when the intercept term was skewed, resulting in a biased ${\hat{\beta}}_{\lambda }$ with low type I error and poor coverage. Since the cprobit model eliminates the intercept terms from the likelihood, it is robust when used with the Box-Cox transformation because it avoids making any distributional assumption on the intercepts.

We summarized the application of our method as a three-step workflow, where Step 1 models the ordered outcomes within each subject to estimate a measure of association between an exposure and a outcome (${\hat{\beta}}_c$) that is invariant to a Box-Cox transformation on the outcome. Leveraging on this invariance property in the subsequent two steps, we scale ${\hat{\beta}}_c$ to provide an appropriate estimate for the exposure effect on the change in the observed (or Box-Cox transformed) outcomes pending the adequacy of the normality assumption. Since ${\hat{\beta}}_c$ is the scaled exposure effect estimate of the linear model that satisfies the normality assumption, different scaling factors are used for the observed and transformed outcomes. The two real datasets that utilize EMRs illustrated the step-by-step application of the method, where the data was first analyzed without transformation in Step 2 after completing Step 1. When the normality assumption is adequate (e.g., the neutrophil study), the estimated exposure effect from Step 2 is reported, otherwise the Box-Cox transformation is used to address the non-normality in Step 3 (e.g., the blood glucose study). Since the cprobit model makes inference by modelling the change within each subject, it estimates the same exposure effect (but with higher variability) as the linear regression model applied to the change in outcomes when no transformation is required. This is illustrated in the neutrophil study, where our proposed approach had similar estimate to the linear regression model but had a wider 95% CI.

In common with the linear regression model for the change in outcomes, our proposed approach has advantages over the REM in handling time-invariant confounding effects. Although the subject-specific intercept in the REM may implicitly account for time-invariant covariates, a correlation between the intercept and the exposure may result in biased estimates when some of the covariates are also confounders. By modeling the difference, our proposed approach can be viewed as a fixed effects approach for analyzing longitudinal data that differs from the REM by allowing for correlation between the subject-specific intercept and exposure [8, 28], hence implicitly controlling for time-invariant confounding effects too. Hence, our proposed approach estimates the within-subject effect of a time-varying exposure, while the between-subject effect can be estimated from a REM that explicitly models these two effects of the time-varying exposure [8]. By modeling the ordered outcomes within each subject with the cprobit model, our proposed approach is robust to misspecified model assumptions but have lower power and higher variability than REM. A similar observation is made by Liu and team [10] when they compared their approach that models ordered outcomes of independent samples with approaches that model the observed outcomes.

Since the outcome of the cprobit model is a binary variable indicating an increase in the outcome for each subject, it is susceptible to common issues faced when analyzing binary responses, e.g., biased estimate due to small sample size, rare events or (quasi-)complete separation [29–31]. The bias resulting from these issues could be alleviated using the Firth’s method [30–32], which is recently implemented for the probit regression model in the R package *brglm* [33]*.* Another limitation shared by the cprobit model and the REM is the high level of uncertainty in estimating *λ* when the sample size is small (*n* = 300), which could result in biased estimates with underestimated SE. However, Box and Cox [3] argued that one can still obtain a reasonable estimate of the effect in these situations by identifying a transformation that overcomes non-normality (e.g., using prior knowledge) and reporting the estimated effect on the transformed outcome, and this is also commonly practiced in real data analyses.

In this paper we considered a common transformation for the two repeated measurements, which may not be applicable for a study where, for example, there is a suspected profound batch effect between the two measurements suggesting the transformation applied to each measurement is different. Wu and Tian [34] proposed a non-parametric transformation that allows each measurement to have a different transformation function from the others. We have considered the use of the Box-Cox transformation in Step 3, and recently, Hothorn and team [35] have proposed a flexible family of non-parametric transformation functions for studies of independent samples of continuous outcomes that is applicable to both positive and negative values. Future work may explore extending their approach for Step 3 of our workflow. Nevertheless, the inference based on ${\hat{\beta}}_c$ from Step 1 of our proposed workflow is valid as long as there exists some (unknown) monotonic transformation where the normality assumption is adequate, hence providing a useful tool for biomarker discovery by making less restrictive assumptions for hypothesis testing [12, 36]. Moreover, our proposed approach alleviates the complications of model building procedures involving the use of transformation on the outcomes to correct model misspecification, which are prevalent in both the traditional Box-Cox transformation [11, 37] and the recent non-parametric approaches [34, 35].

Although we have presented our method as an approach to analyze change in two repeated measurements of a continuous outcome, it also applies to stratified analysis of continuous outcomes for confounder adjustment, with each stratum consisting of a pair of subjects with the same confounding profile. In scenarios with more than two repeated measurements from each subject, or more than two subjects in each stratum, the rank-ordered probit model [38] that generalizes the cprobit model can be considered in the same vein as the stratified analysis of continuous outcomes [12]. Furthermore, our proposed workflow can be extended to other error distributions (e.g., the skewed error distribution considered by Tan and team [12]), where the conventional normality assumption is not expected in some real-life applications [39, 40].

## Conclusions

In this paper, we present a novel application of the cprobit model that provides a robust method for the study of change in a continuous outcome. The method is invariant to any monotonic transformation on the outcome when testing for the presence and direction of the association between the exposure and the outcome, and generally has estimates with good inferential properties for the exposure effect on the (transformed) outcome. Hence, a statistical analysis plan that pre-empts the use of Box-Cox transformation to alleviate non-normality can be easily integrated into the data analysis steps with the method, resulting in a practical and seamless workflow for data analysts.

## Supplementary information

**Additional file 1.** Supplementary tables and figures for “Robust estimation of the effect of an exposure on the change in a continuous outcome”. Includes a detailed visual illustration of the three-step workflow, additional tables for simulation studies and additional figures for real data analysis.

**Additional file 2.** Detailed simulation setup in “Robust estimation of the effect of an exposure on the change in a continuous outcome”. Provides a detailed description of the simulation setup.

**Additional file 3.** Example Usage of Package cprobit. Description of a reproducible example using the cprobit package.

## Data Availability

Data is available upon reasonable request and approval from ethic boards, and can only be shared in the context of an agreed collaboration and subject to a data-sharing agreement to ensure security of the personal data of the study participants. The code for implementing our approach is available as an R package (available from https://github.com/nyilin/cprobit and see Additional file 3 for a reproducible example).

## Abbreviations

BG: Blood glucose
CI: Confidence interval
cprobit: Conditional probit
EMR: Electronic medical records
NUH: National university hospital
REM: Random effects model
SD: Standard deviation
SE: Standard error
WBC: White blood cell
