# Supplementary material for: Robust estimation of the effect of an exposure on the change in a continuous outcome
Source: BMC Med Res Methodol. 2020 Jun 6;20:145. doi: 10.1186/s12874-020-01027-6 (PMC7275496; doi:10.1186/s12874-020-01027-6)

Supplementary tables and figures for "Robust estimation of the effect of an exposure on the change in a continuous outcome"

Tables and figures in the following pages are arranged in the order they are referenced to the in the main text.

Figure S1 Detailed visualization of the three-step workflow of the conditional probit (cprobit) model for the analysis of continuous outcomes and equations corresponding to the underlying statistical framework, with equations numbered as in the text. (REM denotes random effects model).

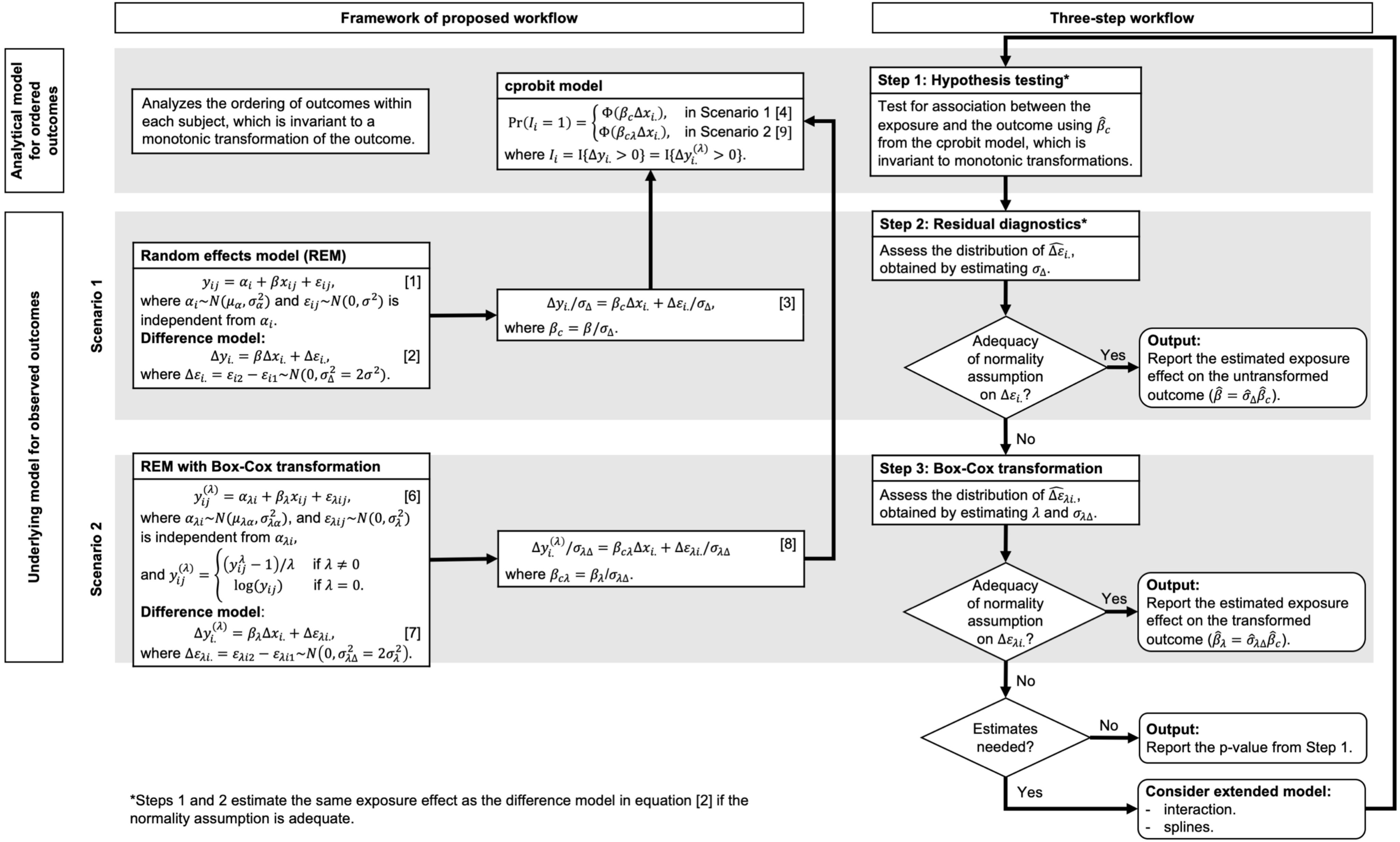

Additional tables for simulation studies

Table S1 Simulation results from the estimation of the linear effect of the predictor (β) in Simulation study 1, where no transformation was required, with varying distributions of intercept terms, sample sizes (n) and effect sizes

**Distribution of intercepts**	**n**	**Method**	$\boldsymbol{\beta = 0}$					$\boldsymbol{\beta = -0.06}$				
			**Bias**	**Emp. SE**	**Mean SE**	**Type I**	**Cov.**	**Bias**	**Emp. SE**	**Mean SE**	**Power**	**Cov.**
Normal	1200	REM	0.000	0.015	0.015	0.052	0.949	0.000	0.015	0.015	0.980	0.950
		cprobit	0.000	0.018	0.018	0.049	0.951	0.000	0.019	0.019	0.901	0.950
	300	REM	-0.001	0.029	0.029	0.051	0.950	0.001	0.030	0.029	0.536	0.945
		cprobit	0.001	0.038	0.037	0.055	0.946	-0.001	0.038	0.038	0.363	0.940
Skewed	1200	REM	0.001	0.015	0.015	0.049	0.952	0.000	0.014	0.015	0.984	0.954
		cprobit	0.001	0.018	0.018	0.049	0.952	0.000	0.018	0.019	0.891	0.954
	300	REM	0.000	0.030	0.029	0.050	0.951	0.000	0.030	0.029	0.545	0.945
		cprobit	0.000	0.038	0.037	0.054	0.946	-0.001	0.037	0.038	0.372	0.953

Emp. SE: Empirical standard error; Mean SE: Mean standard error; Cov.: Coverage; REM: Random effects model; cprobit: conditional probit model

Table S2 Simulation results from the estimation of the linear effect of the predictor (β_λ) in Simulation study 2, where the Box-Cox transformation was applied to the outcome, with varying distribution of intercept terms, sample sizes (n) and effect sizes

Distribution of intercepts	n	λ	Method	$\beta_\lambda = 0$					$\beta_\lambda = -0.06$				
				Bias	Emp. SE	Mean SE	Type I	Cov.	Bias	Emp. SE	Mean SE	Power	Cov.
Normal	1200	1	REM	0.000	0.015	0.015	0.051	0.949	0.000	0.017	0.015	0.981	0.907
			cprobit	0.000	0.019	0.019	0.049	0.951	-0.001	0.022	0.019	0.901	0.905
		$\frac{1}{3}$	REM	0.000	0.015	0.015	0.051	0.949	0.000	0.016	0.015	0.981	0.925
			cprobit	0.000	0.018	0.019	0.049	0.951	-0.001	0.021	0.019	0.901	0.921
		0	REM	0.000	0.015	0.015	0.051	0.950	0.000	0.016	0.015	0.980	0.933
			cprobit	0.000	0.018	0.018	0.049	0.951	-0.001	0.020	0.019	0.901	0.933
	300	1	REM	-0.001	0.031	0.030	0.054	0.947	-0.002	0.036	0.031	0.538	0.908
			cprobit	0.002	0.042	0.039	0.055	0.946	-0.005	0.050	0.040	0.363	0.892
		$\frac{1}{3}$	REM	-0.001	0.030	0.030	0.054	0.947	-0.002	0.034	0.030	0.538	0.926
			cprobit	0.002	0.041	0.039	0.055	0.946	-0.004	0.046	0.039	0.363	0.918
		0	REM	-0.001	0.030	0.030	0.054	0.947	-0.001	0.032	0.030	0.538	0.938
			cprobit	0.001	0.039	0.038	0.055	0.946	-0.003	0.043	0.038	0.363	0.928
Skewed	1200	1	REM	-0.001	0.003	0.003	0.047	0.954	0.048	0.005	0.003	0.959	0.000
			cprobit	0.001	0.019	0.019	0.049	0.952	-0.001	0.022	0.019	0.891	0.909
		$\frac{1}{3}$	REM	-0.002	0.004	0.005	0.047	0.954	0.043	0.006	0.005	0.958	0.001
			cprobit	0.001	0.019	0.019	0.049	0.952	0.000	0.021	0.019	0.891	0.928
		0	REM	-0.003	0.006	0.007	0.044	0.957	0.036	0.007	0.007	0.956	0.004
			cprobit	0.001	0.018	0.019	0.049	0.952	0.000	0.020	0.019	0.891	0.940
	300	1	REM	-0.001	0.009	0.008	0.040	0.961	0.045	0.012	0.008	0.434	0.079
			cprobit	0.002	0.044	0.040	0.054	0.946	-0.006	0.051	0.040	0.372	0.905
		$\frac{1}{3}$	REM	-0.002	0.012	0.011	0.039	0.962	0.040	0.014	0.011	0.432	0.150
			cprobit	0.001	0.042	0.039	0.054	0.946	-0.004	0.046	0.039	0.372	0.927
		0	REM	-0.003	0.015	0.015	0.037	0.963	0.034	0.016	0.015	0.427	0.342
			cprobit	0.001	0.040	0.038	0.054	0.946	-0.003	0.042	0.039	0.372	0.942

Emp. SE: Empirical standard error; Mean SE: Mean standard error; Cov.: Coverage; REM: Random effects model; cprobit: conditional probit model

Table S3 Simulation results from the estimation of the transformation parameter (λ) in Simulation study 2, where the Box-Cox transformation was applied to the outcome, with varying distribution of intercept terms, sample sizes (n) and effect sizes (β_λ).

Distribution of intercepts	n	λ	Method	$\beta_\lambda = 0$					$\beta_\lambda = -0.06$				
				Bias	Emp. SE	Mean SE	Type I / Power	Cov.	Bias	Emp. SE	Mean SE	Type I / Power	Cov.
Normal	1200	1	REM	-0.001	0.076	0.079	0.044	0.956	-0.001	0.082	0.080	0.055	0.945
			cprobit	-0.002	0.099	0.109	0.036	0.964	-0.002	0.103	0.110	0.038	0.962
		$\frac{1}{3}$	REM	0.000	0.037	0.038	1.000	0.957	0.000	0.040	0.039	1.000	0.946
			cprobit	-0.001	0.048	0.053	1.000	0.966	-0.001	0.050	0.053	1.000	0.963
		0	REM	0.000	0.016	0.017	1.000	0.958	0.000	0.018	0.018	1.000	0.946
			cprobit	0.000	0.022	0.024	1.000	0.964	0.000	0.023	0.024	1.000	0.961
	300	1	REM	0.001	0.159	0.160	0.044	0.957	0.007	0.160	0.163	0.044	0.957
			cprobit	0.007	0.207	0.219	0.044	0.957	0.006	0.210	0.222	0.038	0.963
		$\frac{1}{3}$	REM	0.001	0.077	0.077	1.000	0.958	0.004	0.078	0.079	1.000	0.959
			cprobit	0.005	0.100	0.106	1.000	0.954	0.004	0.102	0.108	1.000	0.962
		0	REM	0.002	0.035	0.034	1.000	0.954	0.003	0.035	0.036	1.000	0.957
			cprobit	0.003	0.045	0.047	1.000	0.955	0.003	0.046	0.049	1.000	0.960
Skewed	1200	1	REM	-0.974	0.170	0.072	1.000	0.000	-1.009	0.177	0.074	1.000	0.000
			cprobit	0.005	0.108	0.119	0.032	0.968	0.005	0.110	0.121	0.030	0.970
		$\frac{1}{3}$	REM	-0.470	0.079	0.035	1.000	0.000	-0.489	0.082	0.035	1.000	0.000
			cprobit	0.003	0.051	0.056	1.000	0.969	0.003	0.052	0.058	1.000	0.969
		0	REM	-0.207	0.030	0.015	1.000	0.000	-0.217	0.032	0.016	1.000	0.000
			cprobit	0.002	0.022	0.024	1.000	0.970	0.002	0.023	0.025	1.000	0.967
	300	1	REM	-0.907	0.342	0.151	0.945	0.055	-0.950	0.337	0.155	0.961	0.040
			cprobit	0.008	0.226	0.241	0.032	0.968	0.006	0.229	0.245	0.031	0.970
		$\frac{1}{3}$	REM	-0.436	0.160	0.072	1.000	0.050	-0.460	0.158	0.074	1.000	0.039
			cprobit	0.005	0.108	0.114	1.000	0.967	0.004	0.109	0.116	1.000	0.968
		0	REM	-0.192	0.064	0.032	1.000	0.046	-0.204	0.064	0.033	1.000	0.034
			cprobit	0.004	0.046	0.049	1.000	0.962	0.003	0.047	0.050	1.000	0.965

Emp. SE: Empirical standard error; Mean SE: Mean standard error; Cov.: Coverage; REM: Random effects model; cprobit: conditional probit model

Table S4 Percent of simulation cycles in Simulation study 2 with the Lilliefors test rejecting the adequacy of the normality assumption after applying the Box-Cox transformation to the outcome in the random effects model (REM) and the conditional probit (cprobit) model (REM/cprobit model) for both distributions of intercept terms, with varying sample size (n), transformation parameter value (λ) and effect size (β_λ). Bold font indicates scenarios where the proportion of simulation cycles is much larger than the expected 5% (i.e., the deviation from 5% is greater than or equals to 10%)

		**With normally distributed intercepts**		**With skewed intercepts**	
$\boldsymbol{n}$	$\boldsymbol{\lambda}$	$\boldsymbol{\beta_\lambda = 0}$	$\boldsymbol{\beta_\lambda = -0.06}$	$\boldsymbol{\beta_\lambda = 0}$	$\boldsymbol{\beta_\lambda = -0.06}$
1200	1	3/5	4/3	**82**/5	**81**/5
	1/3	3/5	4/3	**78**/5	**77**/5
	0	3/5	4/3	**63**/5	**62**/5
300	1	5/6	4/6	**26**/6	**24**/5
	1/3	5/6	4/6	**24**/6	**22**/5
	0	5/5	4/6	**18**/6	**17**/5

Additional figures for real data analysis

Figure S2 QQ-plots of the residuals from the linear regression model (panel A), the random effects model (panel B) and the conditional probit (cprobit) model (panel C) in the neutrophil study. The p-values of the Lilliefors test for normality were 0.386, 0.459 and 0.520 for panels A to C.

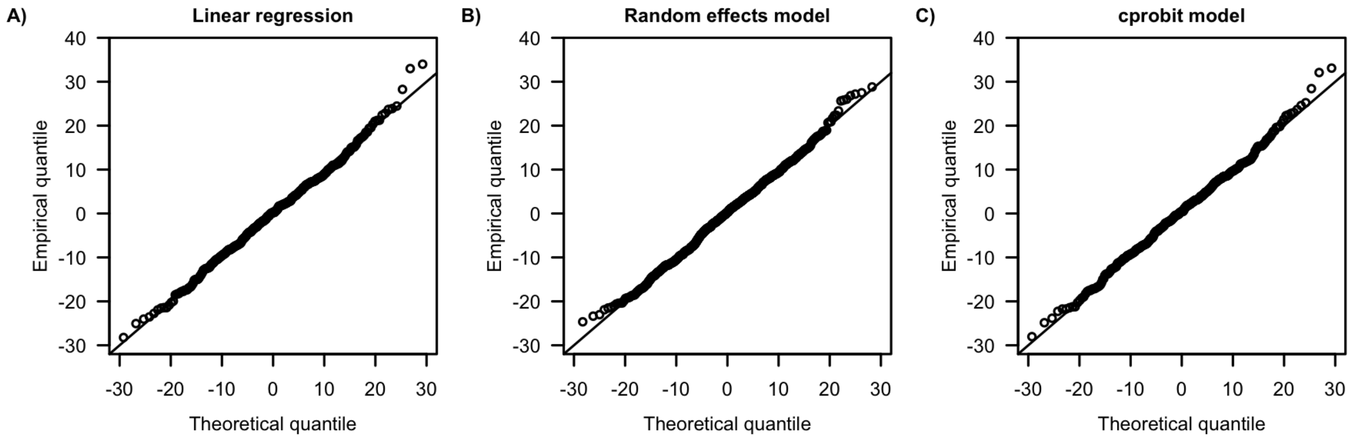

Figure S3 QQ-plot of the residuals from the linear regression model in the blood glucose study. The p-value of the Lilliefors test for normality was < 0.001.

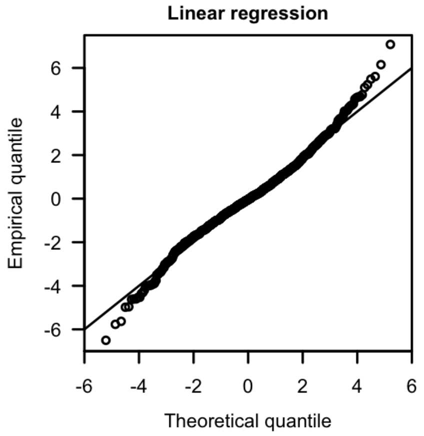

Figure S4 QQ-plots of the residuals from the random effects model (panels A and C) and the conditional probit (cprobit) model (panels B and D) in the blood glucose study, before (top row) and after (bottom row) applying the Box-Cox transformation on the outcome. The p-values of the Lilliefors test for normality were < 0.001, < 0.001, 0.915 and 0.631 for panels A to D.

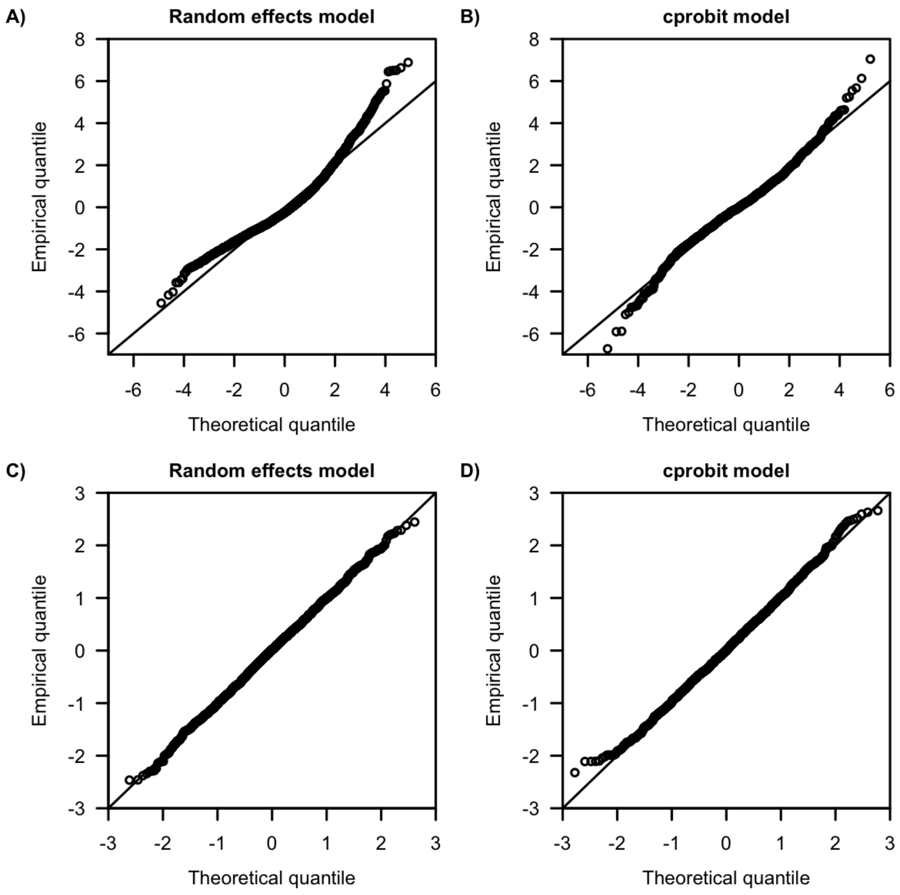

Supplement: Supplementary file 1 — Additional file 1. Supplementary tables and figures for “Robust estimation of the effect of an exposure on the change in a continuous outcome”. Includes a detailed visual illustration of the three-step workflow, additional tables for simulation studies and additional figures for real data analysis. [file 12874_2020_1027_MOESM1_ESM.pdf]
