# Supplementary material for: Robust estimation of the effect of an exposure on the change in a continuous outcome
Source: BMC Med Res Methodol. 2020 Jun 6;20:145. doi: 10.1186/s12874-020-01027-6 (PMC7275496; doi:10.1186/s12874-020-01027-6)
Supplement: Supplementary file 2 — Additional file 2. Detailed simulation setup in “Robust estimation of the effect of an exposure on the change in a continuous outcome”. Provides a detailed description of the simulation setup. [file 12874_2020_1027_MOESM2_ESM.pdf]

Detailed simulation setup in "Robust estimation of the effect of an exposure on the change in a continuous outcome"

The simulation studies are based on a real data from a study of blood glucose (BG) among inpatients at the National University Hospital (NUH), Singapore. The study investigated the effect of the baseline glycemic variability on the change in daily glycemic variability in the two subsequent days (referred to as the first and second follow-up). Daily glycemic variability was measured using the standard deviation (SD) of the BG readings on each day, and the analysis was conducted using data measured from 1200 patients who had at least 3 readings per day, with adjustment for age and gender. The BG data is described in more detail in the "Blood glucose study" section.

Simulation study 1

This study assessed and compared the performance of the REM and the cprobit model when applied to the observed outcome without transformation. The covariates were generated from their respective empirical distributions: for each i-th subject, the cubic root of baseline SD (SD_{0i}) was generated from $N(1.3, 0.3^2)$, age (Age_i) was generated from $N(68.6, 13.6^2)$ and the indicator for being female (Female_i) was generated from $\text{Bernoulli}(0.5)$.

Building on equation [1] of the main text, we generated continuous outcomes for the first and second follow-up of BG variability from the following linear model:

$$y_{ij} = \alpha_i - 0.015t_{ij} + 0.283SD_{0i} + 0.001\text{Age}_i - 0.059\text{Female}_i + \beta t_{ij}SD_{0i} + \varepsilon_{ij}, \qquad \text{[A1]}$$

where t_{ij} is an indicator for being the second follow-up measurement (i.e., $t_{i1} = 0$ and $t_{i2} = 1$), the error terms ε_{ij} were generated from $N(0, \sigma^2 = 0.6^2)$, and the random intercepts α_i were generated from $N(\mu_\alpha = 3.5, \sigma_\alpha^2 = 0.5^2)$, for $i = 1, \dots, n$, and $j = 1, 2$. The values of the parameters used in the simulation were referenced from the REM with Box-Cox transformation applied to the real data. We

also considered random intercept terms with a skewed distribution to assess the potential impact of model misspecification on the intercept terms in the REM, especially when the Box-Cox transformation was used (see Simulation study 2). Specifically, the skewed intercept was generated from $\alpha_i = \alpha_i^* + \mu_\alpha - \sigma_\alpha^2/2$, where α_i^* was generated from a Chi-square distribution with $\sigma_\alpha^2/2$ degrees of freedom to maintain the same mean and variance as the normally distributed intercept.

Simulation study 2

This study assessed and compared the performance of the two methods when the Box-Cox transformation was applied to the outcome. We chose the value for μ_α to ensure the generated y_{ij} from equation [A1] provides positive outcomes for the various λ values prior the Box-Cox transformation (where y_{ij} in Simulation study 1 is equivalent to $y_{ij}^{(\lambda)}$ in Simulation study 2). Hence, we applied the inverse Box-Cox transformation to y_{ij} generated from equation [A1] to obtain the observed continuous outcome prior to the (supposedly unknown) transformation, where $\lambda = 1, 1/3, 0$. Thus, the created data required no transformation, a cubic root transformation and a log transformation on the observed outcomes respectively to satisfy the normality assumption.
