# Supplementary material for: Robust estimation of the effect of an exposure on the change in a continuous outcome
Source: BMC Med Res Methodol. 2020 Jun 6;20:145. doi: 10.1186/s12874-020-01027-6 (PMC7275496; doi:10.1186/s12874-020-01027-6)
Supplement: Supplementary file 3 — Additional file 3. Example Usage of Package cprobit. Description of a reproducible example using the cprobit package. [file 12874_2020_1027_MOESM3_ESM.pdf]

Example Usage of Package cprobit

The cprobit package includes a realistic dataset (`bg_variability`) generated based on the dataset analysed in the blood glucose study in "Robust estimation of the effect of an exposure on the change in a continuous outcome".

This study investigated the effect of the baseline glycemic variability on the change in daily glycemic variability in the two subsequent days (referred to as the first and second follow-up), using data from 1200 subjects. Daily glycemic variability was measured using the standard deviation of the blood glucose readings on each day. Each row in the dataset corresponds to one follow-up measurement from each subject, and the first 10 rows are shown below:

```r
# Firstly, use the following command to install the `cprobit` package from
# Github (package `devtools` required):
# devtools::install_github("nyilin/cprobit")
library(cprobit)
data("bg_variability")
dim(bg_variability)
## [1] 2400    7
knitr::kable(head(bg_variability, 10), digits = c(0, 0, 2, 0, 2, 0, 0))
```

subject_id	case_id	y	t	sd0	age	female
1	1	9.94	0	1.30	77	1
1	2	9.10	1	1.30	77	1
2	1	13.61	0	1.62	64	0
2	2	11.48	1	1.62	64	0
3	1	10.49	0	3.01	34	0
3	2	13.13	1	3.01	34	0
4	1	18.56	0	2.29	49	1
4	2	19.41	1	2.29	49	1
5	1	14.52	0	1.33	56	1
5	2	12.78	1	1.33	56	1

Variables `subject_id = 1, ..., 1200` and `case_id = 1, 2` are identifiers of subjects and follow-ups respectively. Variable `y` denotes the continuous outcome, i.e., the glycemic variability in the first and second follow-ups. Variable `t = 0, 1` is the binary indicator for the second follow-up. Variable `sd0` denotes the time-invariant exposure, i.e., the baseline glycemic variability. Age (`age`) and gender (`female = 1` for female and `female = 0` for male) are two time-invariant confounders.

Equation [13] of the manuscript presents the random effects model assumed when assessing the association between the baseline measurement and the change in the follow-up measurements:

$$y_{ij} = \alpha_i + \beta_1 t_{ij} + \beta_2 sd0_i + \beta_3 t_{ij} sd0_i + \beta_4 age_i + \beta_5 female_i + \varepsilon_{ij}.$$

The effect of interest is β_3, because it is the the coefficient of the time-invariant exposure, `sd0`, in the difference model:

$$\Delta y_i = \beta_1 + \beta_3 sd0_i + \Delta\varepsilon_i,$$

where $\Delta y_i = y_{i2} - y_{i1}$ and $\Delta\varepsilon_i = \varepsilon_{i2} - \varepsilon_{i1}$ (see equation [14] in the manuscript).

The following command implements the three-step workflow for estimating β_3. Note that time-invariant components in the random effects model, i.e., `sd0`, `age` and `female`, are not included in the cprobit model because they are eliminated by working on the difference data.

```
model <- cprobit(formula = y ~ t + t:sd0, dat = bg_variability,
                 index = c("subject_id", "case_id"),
                 transform = NULL, resid_pval_threshold = 0.05)
summary(model, plot = TRUE)
## ## Results from Step 2:
##
## Lilliefors test p-value for normality assumption
##      without transforamtion: 0.011 < 0.050
##
## Estimated coefficients for observed outcome:
##
##          var    est    se ci_lower ci_upper pval
## t          t  0.668 0.341    0.000    1.335 0.05
## t:sd0 t:sd0 -0.265 0.114   -0.488   -0.041 0.02
## ## Results from Step 3:
##
## Box-Cox transforamtion on the outcome:
##
##            var   est    se ci_lower ci_upper pval
## lambda lambda 0.388 0.056    0.279    0.498    0
##
## Lilliefors test p-value for normality assumption
##      after transformation: 0.314 >= 0.050
##
## Estimated coefficients for transformed outcome:
##
##          var    est    se ci_lower ci_upper pval
## t          t  0.129 0.066    0.000    0.258 0.05
## t:sd0 t:sd0 -0.051 0.022   -0.094   -0.008 0.02
```

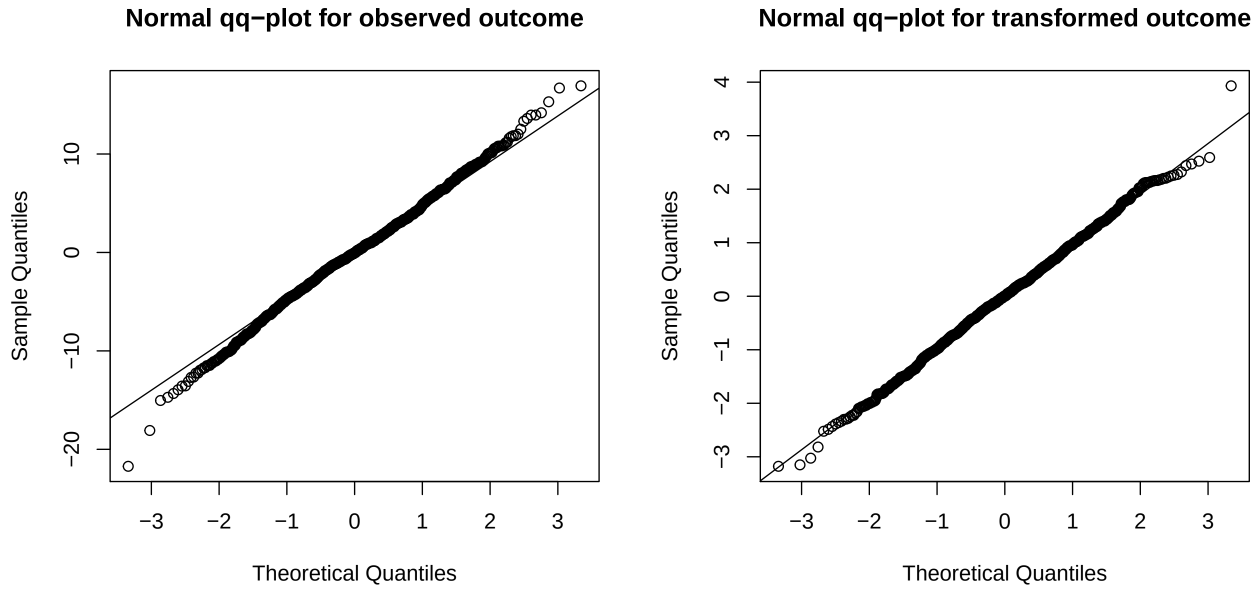


```
table2_row2 <- data.frame(
  Method = "cprobit",
```

```r
  `Linear effect etimate (95% CI)` =
    sprintf("%.3f (%.3f, %.3f)", model$Step3$coef$est[2],
            model$Step3$coef$ci_lower[2], model$Step3$coef$ci_upper[2]),
  `Trasformation parameter estimate (95% CI)` =
    sprintf("%.2f (%.2f, %.2f)", model$Step3$transformation$est,
            model$Step3$transformation$ci_lower,
            model$Step3$transformation$ci_upper),
  check.names = FALSE
)
knitr::kable(table2_row2, align = c("l", "c", "c"))
```

Method	Linear effect etimate (95% CI)	Trasformation parameter estimate (95% CI)
cprobit	-0.051 (-0.094, -0.008)	0.39 (0.28, 0.50)

By specifying `transform = NULL`, Lilliefors test of residuals in Step 2 is used to identify the need for a Box-Cox transformation of the outcome. With Lilliefors p-value in Step 2 less than the selected threshold of 0.05, the Box-Cox transformation on the outcome in Step 3 is used to address non-normality, with estimated transformation parameter 0.39 (95% CI: 0.28, 0.50), and the Lilliefors test (p-value = 0.314) suggests the adequacy of the normality assumption after transformation. The estimated exposure effect on the transformed outcome is -0.051 (95% CI: -0.094, -0.008). Residual qq-plots corroborated the need for the Box-Cox transformation. These findings are similar to the results from the blood glucose study.
